# COP9 Signalosome Subunit *SlCSN5-3* Positively Regulates Resistance to Gray Mold Disease in Tomato (*Solanum lycopersicum*) Through Jasmonic Acid Pathway

**DOI:** 10.3390/biology14121635

**Published:** 2025-11-21

**Authors:** Rui Lv, Hecheng Sun, Fulei Mo, Shusen Liu, Zhao Liu, Xiuling Chen, Yuxin Liu, Aoxue Wang

**Affiliations:** 1 College of Horticulture and Landscape Architecture, Northeast Agricultural University, Harbin 150030, China; lvrui34324080@163.com (R.L.); 13940490565@163.com (H.S.); chenx@neau.edu.cn (X.C.); 2 College of Life Sciences, Northeast Agricultural University, Harbin 150030, China; neaumfl@163.com; 3 School of Life Science and Technology, Harbin Institute of Technology, Harbin 150001, China; 4 Shandong Shouguang Sanmu Seedling Co., Weifang 250013, China; shusenliu2023@163.com (S.L.); sanmuliuzhao@163.com (Z.L.); 5 Key Laboratory of Biology and Genetic Improvement of Horticultural Crops (Northeast Region), Ministry of Agriculture and Rural Affairs, Harbin 150030, China

**Keywords:** CSN5, *Botrytis cinerea*, tomato, JA, VIGS

## Abstract

CSN5 is one of the subunits of the COP9 signalosome, which can regulate plant life activities by participating in the ubiquitination process. It is also positively correlated with JA signal intensity in plants and regulates JA-dependent plant defense responses. In this study, a total of three *CSN5* family genes were identified in tomatoes and a systematic bioinformatics analysis was performed to clarify their similarities and differences. In addition, by analyzing the publicly available transcriptome data and qRT-PCR experiments, it was found that *SlCSN5-2* and *SlCSN5-3* were up-regulated under gray mold disease stress, and the potential key gene *SlCSN5-3* was up-regulated most significantly. Silencing *SlCSN5-3* in tomatoes resulted in reduced resistance to gray mold disease, and it was clear that *SlCSN5-3* positively regulates tomatoes’ resistance to gray mold disease through the JA pathway.

## Simple Summary

Tomatoes are widely cultivated as a vegetable crop around the world. Gray mold disease, as a disease that can occur in tomatoes, not only affects the yield and fruit quality of tomatoes but also causes direct death in severe cases. It is necessary to screen the resistance genes and analyze the regulatory mechanism of their resistance to gray mold disease, so as to improve the pertinence of disease resistance breeding. In this study, the resistance gene to gray mold disease in cultivated tomatoes was identified using bioinformatics methods, and the molecular regulatory mechanisms of this gene regulating tomatoes’ resistance to gray mold disease were analyzed, which provided a new perspective for understanding the regulatory mechanisms of plants’ resistance to disease.

## 1. Introduction

COP9 signalosome in plants was first identified in Arabidopsis (*Arabidopsis thaliana*). The structure of this complex protein is highly conserved, which can inhibit light morphogenesis and is a regulator of light control during plant development [1,2,3]. A total of eight subunits together make up the COP9 signalosome, and these subunits are designated CSN1-8 according to their molecular mass. Subunits CSN5 and 6 both contain the MPN domain, and the other six subunits contain variable amounts of the proteasome, COP9 signalosome domain, and eIF3 domain [2]. The MPN domain is essential for maintaining the stability of the entire protein complex, so the CSN5 and 6 subunits containing the MPN domain are considered to be the two core subunits of the COP9 signalosome [4,5].

As the active center of the COP9 signalosome, CSN5 can not only exert peptidase activity in the form of a monomer but also functions as a complex. CSN5 participates in the UPS process, which finely manages protein production and breakdown and affects all aspects of the plant life cycle. For example, when a *CSN5* antisense expression vector was transferred into transgenic Arabidopsis, overexpressing the pea (*Pisum sativum*) AUX/IAA protein IAA6, psIAA6 was more stable and attenuated auxin signaling in the transgenic plants. This indicates that the degradation of the Arabidopsis SCFTIR1 substrate psIAA6 is promoted by the interaction between CSN and SCFTIR1 [6]. In Arabidopsis, two distinct genes (*CSN5A/B*) encode the CSN5 protein. Mutations in *CSN5A* can inhibit lateral root and root hair formation, as well as flower development [7]. In addition, CSN5 subunits also show different regulatory roles in seed germination [8].

In addition to regulating plant life activities by participating in the ubiquitination process, the CSN5 expression level is positively correlated with the JA signal intensity in plants and regulates JA-dependent plant defense responses. For example, silencing *CSN5* prevents JA synthesis and reduces resistance to insects and fungi in plants [9].

Most studies of COP9 signalosomes and their subunits in organisms focus on protein structure analysis and functional verification, but the identification and analysis of this family in the same species are rarely reported. Gene family studies of the systematic COP9 signalosome and its subunits have not been reported in plants: only in animals (*Bactrocera dorsalis* and *Penaeus monodon*) [10,11]. In this study, the CSN5 family members of the COP9 signalosome subunit were identified on a genome-wide scale in tomatoes by using a bioinformatics approach for the first time. The locations of the tomato *CSN5* family genes on chromosomes, as well as their structures, were visualized, and the physicochemical properties and structures of the proteins encoded by these genes were also characterized. The phylogeny of the CSN5 family proteins in different species and the collinearity of the *CSN5* family genes was analyzed to understand the evolution of the *CSN5* plant family. *Cis*-acting elements contained in the promoter regions of these tomato *CSN5* family genes were also identified and statistically classified. In addition, the changes in the expression of tomato *CSN5* genes under gray mold disease stress were clarified by transcriptome data analysis and qRT-PCR experiments, and the function of *SlCSN5-3* in resistance to gray mold disease was verified by silencing the *SlCSN5-3* gene. This study elucidated the evolutionary and functional divergence of *CSN5* family genes in tomatoes, providing new insights into the mechanisms of plant disease resistance.

## 2. Materials and Methods

### 2.1. Plant Materials and Microbial Stress

In this study, the tomatoes used for detecting gene expression and the VIGS experiment were cultivated tomatoes of the Ailsa Craig variety (*Solanum lycopersicum* L., LA2838A). Seeds were sown in moist, fertile seedling soil and grown in a plant incubator (HPG-320BX, Harbin Donglian Co., Ltd., Harbin, China). The parameters of the plant incubator were set as follows: Light 16 h, light intensity 150 μM photons m^−2^s^−1^, temperature 28 °C. Dark 8 h, temperature 23 °C. The relative air humidity was 85% throughout the day (favorable for inoculation of *B. cinerea*). When tomatoes reached 28-day seedling age, they were inoculated with *B. cinerea*. *B. cinerea* used in this study was obtained from tomatoes with gray mold disease from the Xiangyang Farm, Northeast Agricultural University of China. The pathogen was isolated from the infected tomatoes and incubated. The results were verified by sequencing and were consistent with the strains collected by NCBI (NCBI accession number: 40559).

*B. cinerea* strains were inoculated on solid PDA medium (LBP11123, Laibo Biochemical Technology Co., Ltd., Shanghai, China), and when *B. cinerea* mycelium filled the surface of the medium (approximately 7 days of incubation), the *B. cinerea* medium was rinsed with sterile water to collect spores from *B. cinerea*, and 25 mL of sterile water was used for rinsing each Petri dish. The spore suspension of *B. cinerea* was sprayed evenly on the front and back sides of the tomato leaves, using a watering can (the spore concentration was calculated by the hemocytometer as follows: 4 × 10^6^/mL), and the tomato plants continued to grow in a plant incubator.

At 0, 1, 6, 12, 24, 60, and 120 h after gray mold disease stress treatment [12], 0.5 g of tomato leaves were collected and put into a sterilized 1.5 mL centrifuge tube, using sterilized forceps for total RNA extraction and qRT-PCR to analyze the expression patterns of *CSN5* family genes in tomatoes under gray mold disease stress.

### 2.2. Identification of Tomato CSN5 Family Members

The publicly available tomato genome data and its annotation (SL4.0) were obtained from the Phytozome database (https://phytozome-next.jgi.doe.gov/, accessed on 22 January 2025). The sequences of CSN5 proteins that have been validated in Arabidopsis (Protein number: AT1G22920.1) were obtained from the TAIR website (https://www.arabidopsis.org/, accessed on 22 January 2025). The CDS sequences of all tomato genes were extracted and translated into protein sequences, the Arabidopsis CSN5 protein sequence was submitted to the tomato protein sequences for BLAST to search for proteins similar to the Arabidopsis CSN5 protein in tomato using TBtools (v2.138), and the E-value of BLAST search was set to 1 × 10^−5^. The HMMs of the CSN5 proteins (PF18323) were downloaded from the InterPro website (https://www.ebi.ac.uk/interpro/, accessed on 24 January 2025) and submitted to TBtools (v2.138) together with the tomato protein sequences for the HMM search [13]. The intersection of the search results of BLAST and HMM were taken as the *CSN5* family members in tomatoes. Finally, the domains of the identified tomato CSN5 family proteins were examined one by one.

### 2.3. Analysis of Chromosomal Localization and Protein Physicochemical Properties of Tomato CSN5 Family Members

The information for all tomato chromosomes (number, length, and density of gene distribution) was extracted from the genome annotation file. The location information of the *SlCSN5* family genes was obtained according to gene number. TBtools software was used to visualize the chromosomal localization of the tomato *CSN5* family genes. Tomato CSN5 family protein sequences were extracted and submitted to the Expasy website for analysis of their physicochemical properties (https://www.expasy.org/, accessed on 3 February 2025).

### 2.4. Gene Structure Analysis and Protein Conserved Motif and Domain Analysis of Tomato CSN5 Family Members

The exon and intron information of the *CSN5* family genes in tomatoes were extracted from the tomato genome annotation file. To analyze the conserved motifs of the CSN5 family proteins, the sequences of these proteins were submitted to the MEME website (https://meme-suite.org/, accessed on 10 February 2025), and the conserved motif parameters were set to default (10). The conserved domains were identified using the Web CD-Search tool on the NCBI website (https://www.ncbi.nlm.nih.gov/Structure/, accessed on 10 February 2025). The above analysis results were visualized using TBtools software.

### 2.5. Phylogenetic Analysis

The sequences of all known CSN5 proteins in Arabidopsis were obtained from the TAIR website. Published CSN5 protein sequences from other species (plants) were obtained by a keyword search from the protein database of the NCBI website (https://www.ncbi.nlm.nih.gov/protein/?term=, accessed on 1 March 2025). The above protein sequences were used to construct a phylogenetic tree of the CSN5 plant family proteins. The Poisson model was used to build a tree, and the data alignment rules use Clustal W (TBtools, v2.138). The neighbor-joining method was used to analyze the phylogeny of CSN5 family proteins in tomatoes and other plants. The phylogenetic tree of CSN5 plant proteins was visualized using the ITOL website (https://itol.embl.de/, accessed on 3 March 2025).

### 2.6. Collinearity and Synteny Analysis of CSN5 Family Genes

The genome file and its annotation file (SL4.0) were submitted to TBtools software to analyze the collinearity of *SlCSN5* family members in tomato. The genome and annotation files of Arabidopsis and potato (*Solanum tuberosum*) were downloaded from the Ensembl Plants website (https://plants.ensembl.org/, accessed on 4 March 2025). The genome and annotation files of Arabidopsis and potato were submitted together with the tomato genome and annotation file to TBtools software to analyze the synteny between *SlCSN5* genes and *CSN5* genes in other plants, respectively.

### 2.7. Analysis of Cis-Acting Elements in Promoter

Sequences 2000 bp upstream of the start codon of all tomato *CSN5* family genes were extracted from the tomato genome file. The above sequences were submitted to the PlantCARE website (https://bioinformatics.psb.ugent.be/webtools/plantcare/html/, accessed on 6 March 2025) for the identification of *cis*-acting elements. The identification results were counted and visualized using TBtools software.

### 2.8. Transcriptome Data Analysis of Tomato CSN5 Family Genes Under Gray Mold Disease Stress

The transcriptome sequencing data of tomatoes under gray mold disease stress were obtained from already published work from our laboratory [14]. Based on the gene number of the *SlCSN5* family genes, the FPKM of *SlCSN5* members under gray mold disease stress at 0 h, 1 h, and 24 h were extracted from the transcriptome data. These FPKM values were visualized into heat maps using TBtools software.

### 2.9. Total RNA Extraction and qRT-PCR Analysis

The total RNA used in this study was extracted from tissues from Ailsa Craig, using the Trizol method. Reverse transcription reactions were performed using the total RNA as a template, using HiScript III RT SuperMix for qPCR (+gDNA wiper) (Vazyme Biotech Co., Nanjing, China). Primer designs from the online tool on the NCBI website (https://www.ncbi.nlm.nih.gov/tools/primer-blast/, accessed on 5 April 2025) and all qRT-PCR primers are listed in Table S1. The qRT-PCR analysis was performed using qTOWER3G (Analytical Instruments Jena GMBH, Jena, Germany) and ChamQ Universal SYBR qPCR Master Mix (Vazyme Biotech Co., Nanjing, China). The temperature and time of the reaction referenced the instruction and primer Tm values. *β-actin* in tomatoes was chosen as the reference gene, and the relative expression was calculated using the 2^−∆∆Ct^ method [15].

### 2.10. Silencing SlCSN5-3 in Tomatoes by VIGS

The CDS sequence of the *SlCSN5-3* gene was submitted to the VIGS Tool on the SGN website (https://solgenomics.net/, accessed on 6 April 2025) and a 300 bp target sequence was designed. Primers (Table S1) were designed according to the target and vector sequences and PCR amplification was performed (A600, Hangzhou Langji Scientific Instrument Co., Ltd., Hangzhou, China). The target sequences were ligated into the pNC-pTRV2 empty vector using the Nimble Cloning method [16]. The recombinant VIGS vector pTRV2-SlCSN5-3 was sequenced by the Sanger method and used in subsequent experiments. The recombinant VIGS vector, empty VIGS vector, positive control VIGS vector, and vector pTRV1 were transformed into *Agrobacterium tumefaciens* (GV3101), respectively. In this study, three groups of VIGS experiments were set up, namely a *SlCSN5-3* silencing group (pTRV1 + PTRV2-SlCSN5-3), a negative control group (pTRV1 + pTRV2), and a positive control group (pTRV1 + PTRV2-PDS). The bacterial solution containing the vectors described above was injected into the leaves of 21-day-old tomato seedlings, using the needle-free syringe with the method proposed by Chen et al. [17]. Seedlings which transformed with the *Agrobacterium tumefaciens* solution were protected from light for one day and then cultured in a plant incubator at a regular temperature and with light, as described in Section 2.1 above. After 17 days, the silencing efficiency of the *SlCSN5-3* gene was measured by qRT-PCR (in Section 2.9 above).

### 2.11. Phenotype Observation, Disease Statistics, and Physiological Indicators

The control tomatoes and *SlCSN5-3*-silenced tomatoes were subjected to gray mold disease stress treatment, according to the method in Section 2.1 of this article. The phenotype of tomatoes was observed 120 h after inoculation with the fungus. Photoshop (version 19.0) was used to calculate the area of the lesion in the phenotype image, based on the scale in the phenotype image. The number of pixels S1 of 1 cm^2^ in the figure and the number of pixels S2 in the lesion area were counted and the area of the lesion was (cm^2^) = S2/S1. At this time, we calculated the rate of diseased leaves and the average plaque area. Diseased leaf rate = number of leaves with visible disease spots/total number of leaves in this seedling. The mean diseased spot area was calculated using the weighing method. The 10 mm × 10 mm tomato leaves were taken with a standard scale and weighed out by weight W1, using an analytical balance. The diseased spot area of leaves was taken and the weight W2 was measured. The total number of leaves to be measured is denoted as N. The mean diseased spot area was (mm^2^) = [(100 × W2)/W1]/N [18].

Trypan blue staining experiment: 10 mg of trypan blue (Beijing Coolaibo Technology Co., Ltd., Beijing, China), 40 mL of ethanol (Tianjin Fuyu Fine Chemical Co., Ltd., Tianjin, China), 10 mL of lactic acid (Tianjin Fuyu Fine Chemical Co., Ltd., Tianjin, China), 10 mL of glycerol (Tianjin Fuyu Fine Chemical Co., Ltd., Tianjin, China), and 10 mL of phenol (Tianjin Fuyu Fine Chemical Co., Ltd., Tianjin, China) were added together to 10 mL of distilled water and dissolved by heating in a boiling water bath. Tomato leaves were added to the staining solution in a boiling water bath for 3 min and then cooled at room temperature. The stained leaves were fixed using 50% chloral hydrate (Tianjin Kemio Chemical Reagent Co., Ltd., Tianjin, China) and then observed. The purity of the above reagents is analytically pure. The in situ accumulation of H_2_O_2_ and O_2_^−^ was determined by DAB [19] and NBT staining [20], respectively.

The leaf samples of the control tomatoes and the *SlCSN5-3* silenced tomatoes under gray mold disease stress for 0, 1, 6, 12, 24, 60, and 120 h were collected to detect the activities of CHI, PAL, GLU, and PPO. The activities of the aforementioned enzymes were detected by using the commercial kits, JDZM-1-G, PAL-1-Y, GA-1-Y, and PPO-1-Y (Suzhou Keming Biotechnology Co., Suzhou, China), respectively. Enzyme activity assays were performed according to the manufacturer’s instructions.

### 2.12. Hormone Content and Expression of Core Genes in Their Pathway

The leaf samples of the control tomatoes and the *SlCSN5-3* silenced tomatoes under gray mold disease stress for 0, 1, 6, 12, 24, 60, and 120 h were collected to detect the content of SA and JA and the expression of core genes in their pathway. The SA and JA contents were detected by using commercial kits and SaA-4-Q and JA-4-Q (Suzhou Keming Biotechnology Co., Suzhou, China), respectively. The assay was performed using the method in the instruction manual. SA and JA pathway core genes were detected using the qRT-PCR mentioned in Section 2.9 of this article, and primers were listed in Table S1.

### 2.13. Statistical Analyses

IBM SPSS software (version 22) was used for the statistics of the data in the article. One-way ANOVA and two-way ANOVA were used to analyze the significance of the data. The means were separated using Fisher’s protected LSD test at the 5% level of probability in two-way ANOVA, and the *t*-test was used in a one-way ANOVA.

### 2.14. Experiment Repetition

In this study, CHI, PAL, GLU, and PPO activities, SA and JA content, and all qRT-PCR experiments were performed with three biological and mechanical replicates per sample. Three repeated measurements were performed for each leaf sample when measuring the area of diseased leaf spots.

## 3. Results

### 3.1. Identification of CSN5 Family Genes in Tomato

The known Arabidopsis CSN5 protein was submitted to the total protein sequence obtained from the above translation to BLAST out proteins with similar sequences. The HMM model of the CSN5 protein was downloaded from the InterPro website and submitted to all tomato protein sequences for HMM identification. The BLAST search results and HMM identification results were combined as preliminary results, and three *CSN5* genes were finally identified in tomatoes after checking the domains one by one (Table S2).

### 3.2. Chromosomal Localization of CSN5 Family Genes in Tomatoes and Physicochemical Properties of Encoded Proteins

**Figure 1:**
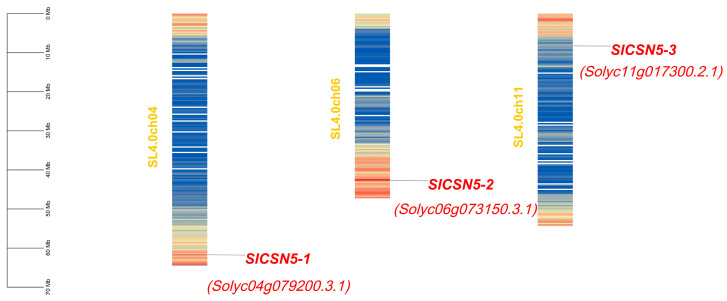
Chromosomal localization analysis of *CSN5* family genes in tomato. The scale line indicates the length of the chromosome. The filled color of the chromosome in the figure indicates the high or low gene density of that chromosome. Red filling indicates a higher density of genes in this region, and blue filling indicates a vs.

The tomato *CSN5* family genes were named *SlCSN5-1*, *SlCSN5-2*, and *SlCSN5-3*, based on their location on the chromosome. The chromosomal localization results of the tomato *CSN5* family genes showed that *SlCSN5-1*, *SlCSN5-2*, and *SlCSN5-3* were located on chromosomes 4, 6, and 11, respectively. All of them were distributed at both ends of the chromosomes, indicating that the tomato *CSN5* family genes may have high expression activity (Figure 1). The amino acid lengths of *SlCSN5-1*, *SlCSN5-2*, and *SlCSN5-3* were 312, 367, and 367; the molecular weights were 34.71, 40.84, and 40.84 KDa; the isoelectric points were 6.17, 5.06, and 4.99, respectively (Table S2).

### 3.3. Structural Analysis of Tomato CSN5 Family Genes and Analysis of Conserved Motifs and Domains of Their Encoded Proteins

**Figure 2:**
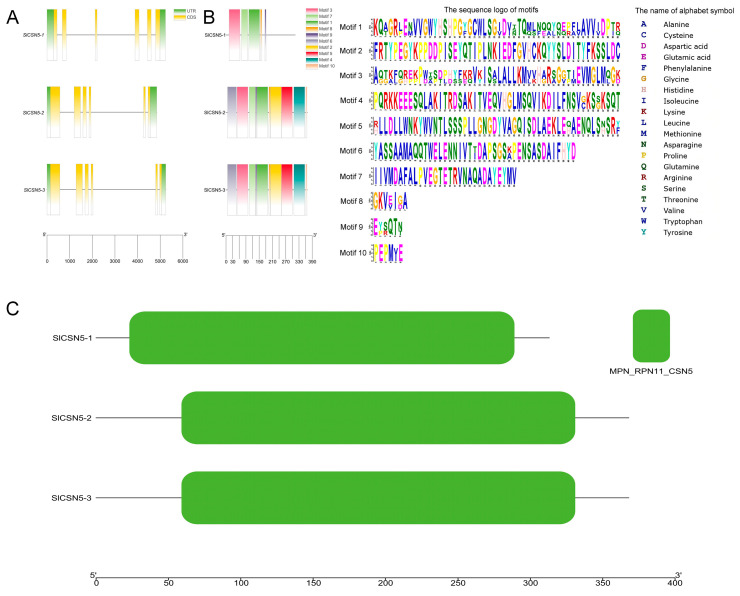
(**A**) Visualization of the gene structure of the tomato *CSN5* family. Green squares represent UTR regions, yellow squares represent CDS sequences, black lines represent introns, and lower scale lines represent gene lengths. (**B**) Conserved motif analysis of the tomato CSN5 family proteins. Different colored squares indicate different motifs, and the lower scale indicates the length of the protein. (**C**) Visualization of conserved domains of the tomato CSN5 family proteins. The lower scale indicates the length of the protein.

The structure of tomato *CSN5* family genes was analyzed according to tomato genome annotation files, and the results showed that all tomato *CSN5* family genes contained introns (Figure 2A). The results of the conserved motif identification showed that SlCSN5-1, SlCSN5-2, and SlCSN5-3 contained 5, 10, and 10 conserved motifs, respectively. In addition, motifs 1, 3, 7, 8, 9 were identified in all CSN5 family proteins (Figure 2B). The results of the conserved domain analysis showed that all the CSN5 family proteins contained only the MPN_RPN11_CSN5 domain, which indicated that the tomato CSN5 family protein sequences were highly conserved (Figure 2C).

### 3.4. Phylogenetic Analysis of CSN5 Family Proteins

**Figure 3:**
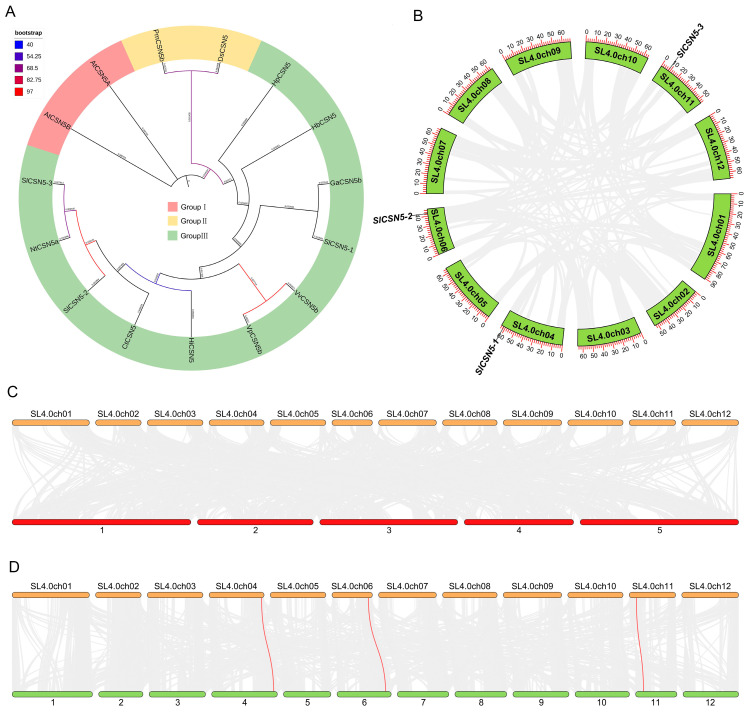
(**A**) Phylogenetic tree of CSN5 family proteins in tomatoes and other plants. (**B**) Collinearity among *CSN5* family genes in tomato. The red line represents the collinearity of *CSN5* genes (no collinearity between three tomato *CSN5* genes), and the gray line represents the collinearity between other genes. (**C**) Synteny of tomato and Arabidopsis *CSN5* members. Tomato chromosomes are shown at the top and Arabidopsis chromosomes at the bottom. The red line represents the synteny of *CSN5s* (no collinearity between tomatoes and Arabidopsis *CSN5* genes), and the gray line represents the synteny of other genes. (**D**) The synteny of tomato and potato *CSN5* members. Tomato chromosomes are shown at the top and potato chromosomes are at the bottom. The red line represents the synteny of *CSN5* genes, and the gray line represents the synteny between other genes.

To understand the evolution and typing of CSN5 family proteins in plants, the CSN5 protein sequence in *Arabidopsis thalianas*, *Carthamus tinctorius*, *Vitis pseudoreticulata*, *Vitis vinifera*, *Nicotiana tabacum*, *Hypericum perforatum*, *Handroanthus impetiginosus*, *Gossypium australe*, *Hevea brasiliensis*, *Dunaliella salina*, and *Panicum miliaceum* were used together with those of tomato CSN5 family proteins to construct phylogenetic trees. Phylogenetic analysis showed that the CSN5 proteins were divided into three groups, with all Arabidopsis CSN5 proteins in Group I, PmCSN5b and DsCSN5 in Group II, and CSN5 in tomatoes and other plants in Group III. This indicates that the protein sequence of Arabidopsis CSN5 is highly conserved and unbranched during evolution, and the CSN5 proteins in tomatoes and other plants may come from the same ancestor and have then evolved in different directions (Figure 3A).

### 3.5. Collinearity Analysis of CSN5 Family Genes

To understand the duplication events of tomato *CSN5* family genes, collinearity analysis was performed on the three identified tomato *CSN5* genes. The results showed that there was no collinearity between the three tomato *CSN5* genes (Figure 3B). The synteny between tomatoes’ *CSN5* and other plants’ *CSN5* genes was also analyzed to understand the evolutionary mechanism of *CSN5* genes. The results showed that there was no synteny between the tomato *CSN5* family genes and Arabidopsis *CSN5* family genes (Figure 3C). There were three synteny relationships between tomato *CSN5* family genes and potato *CSN5* family genes (Figure 3D): *SlCSN5-1* with *PGSC0003DMT400020708*, *SlCSN5-2* with *PGSC0003DMT400069203*, and *SlCSN5-3* with *PGSC0003DMT400023896*. This result may be due to the high evolutionary conservation of CSN5 family members in Arabidopsis (Figure 3A) and the close relationship between tomatoes and potatoes.

### 3.6. Analysis of Cis-Acting Elements in the Promoters of Tomato CSN5 Family Genes

**Figure 4:**
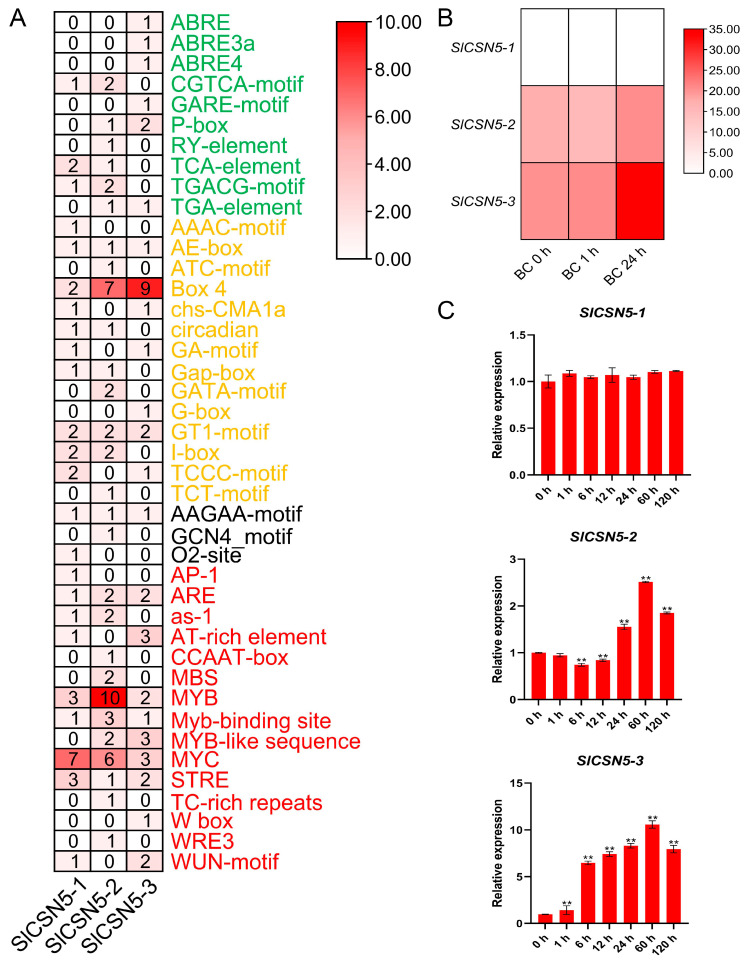
(**A**) The *cis*-acting elements in the promoters of the tomato *CSN5* family genes. Visual heat maps were drawn using TBtools software. The green font color of the element name indicates that the element belongs to the hormone response element, the orange font color of the element name indicates that the element belongs to the light response element, the black font color of the element name indicates that the element belongs to the plant growth and development type element, and the red font color of the element name indicates that the element belongs to the stress response element. (**B**) Heat map of transcriptome data of the tomato *CSN5* family genes under gray mold disease stress. Heat maps were visualized using TBtools software. The darker red color of the heatmap indicates higher expression levels. (**C**) Relative expression of tomato *CSN5* genes under gray mold disease stress. Error lines represent the standard error of the mean of the data from triplicate experiments. ** denotes *p* < 0.001 (*t*-test).

Sequences up to 2000 bp upstream of the start codon of all tomato *CSN5* family genes were extracted and used to identify *cis*-acting elements. Statistical analysis and visualization of the results showed that a total of 42 *cis*-acting elements were found in the tomato *CSN5* family genes, which could be classified into four categories: hormone response, light response, plant development, and stress response. Plant development-type elements, such as AAGAA-motif, were prevalent in the promoters of most genes. These elements were also identified in the promoters of the tomato *CSN5* family genes, but the number of these types of elements is relatively small. In the promoters of the tomato *CSN5* family genes, many kinds of hormone response, light response, and stress response elements were identified, such as ABA element ABRE, light response element Box 4, and the MYB transcription factor binding element that had been repeatedly confirmed to be closely related to stresses in plants. These results indicate the diversity of gene functions in the tomato *CSN5* family (Figure 4A).

### 3.7. Transcriptome Data and qRT-PCR Analysis of Tomato CSN5 Family Genes Under Gray Mold Disease Stress

The transcriptome data of published work from our laboratory were analyzed to obtain a preliminary understanding of the expression of the tomato *CSN5* family genes under gray mold disease stress. The results showed that no FPKM value of *SlCSN5-1* was detected in the transcriptome data, and the expression of *SlCSN5-2* first decreased and then increased under gray mold disease stress, while the expression of *SlCSN5-3* was significantly increased (Figure 4B).

The qRT-PCR analysis of the tomato *CSN5* family genes under gray mold disease stress were performed to gain insight into their expression patterns. The results showed that the expression of *SlCSN5-1* was not significantly changed under gray mold disease stress. The expression of *SlCSN5-2* first decreased slightly, increased at 24 h of stress, and increased to the maximum value at 60 h of stress (2.6-fold), then the expression decreased. *SlCSN5-3* expression was significantly increased under gray mold disease and peaked (over 10-fold) at 60 h of stress, then the expression decreased (Figure 4C). Although the expression of *SlCSN5-2* and *SlCSN5-3* increased under gray mold disease stress, the expression of *SlCSN5-3* increased more significantly than that of *SlCSN5-2*, and it did not show a downward trend in the early stage of stress, so it was hypothesized that the SlCSN5-3 might perform a function within the tolerance of tomatoes to gray mold disease, and *SlCSN5-3* was assumed to be a potential key gene for functional study.

### 3.8. Silencing of SlCSN5-3 in Tomatoes by VIGS

**Figure 5:**
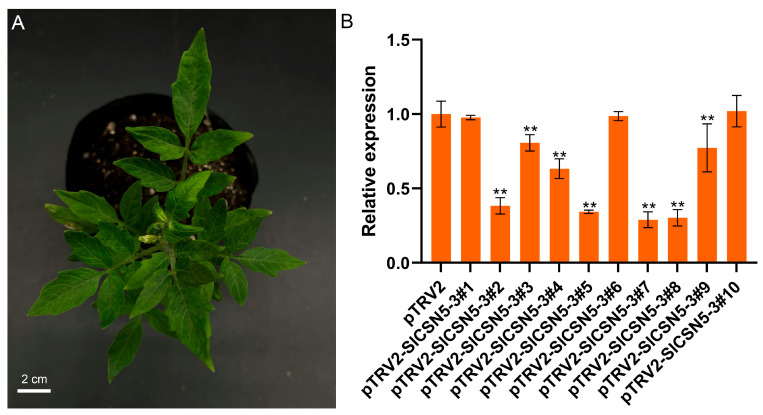
(**A**) The VIGS positive control tomatoes showed fading and albinism symptoms 17 days after injection of the bacterial solution. (**B**) Silencing efficiency of *SlCSN5-3* in tomatoes. Error lines represent the standard error of the mean of the data from triplicate experiments. ** denotes *p* < 0.001 (*t*-test).

The *SlCSN5-3* was silenced in Ailsa Craig, using VIGS to understand the function of *SlCSN5-3* in gray mold disease tolerance. *Agrobacterium tumefaciens* solution containing negative control, positive control, and an *SlCSN5-3* silencing vector was transferred into the cotyledon of 21-day-old tomato seedlings. The seedlings transferred to the *Agrobacterium tumefaciens* solution were protected from light for one day and planted in a plant incubator in a conventional environment. In the positive control group, the tomato leaves showed symptoms of fading and albinism 17 days after injection with *Agrobacterium tumefaciens* solution, indicating that the *Agrobacterium tumefaciens* solution containing the vector injected into tomatoes played a role (Figure 5A). The expression of *SlCSN5-3* in the leaves of the negative control tomatoes and *SlCSN5-3* silenced tomatoes was detected using qRT-PCR, and the results showed that the expression of *SlCSN5-3* in most *SlCSN5-3* silenced tomatoes was significantly lower than in the control group (Figure 5B). This indicates that *SlCSN5-3* was successfully silenced in tomato. The tomatoes with higher silencing efficiency were selected and used for subsequent experiments (pTRV2-SlCSN5-3#2, 3, 4, 5, 7, 8).

### 3.9. Silencing of SlCSN5-3 Reduced Tomatoes’ Resistance to Gray Mold Disease

**Figure 6:**
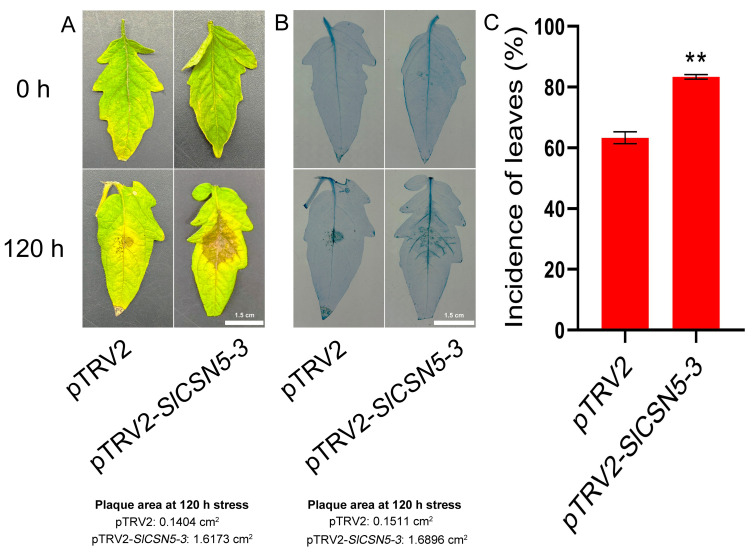
(**A**) Observation of the leaf phenotype of *SlCSN5-3* silenced tomatoes under gray mold disease stress for 120 h (inoculum: 4 × 10^6^/mL spore concentration). (**B**) Trypan blue staining of *SlCSN5-3* silenced tomatoes under gray mold disease stress for 120 h (inoculum: 4 × 10^6^/mL spore concentration). (**C**) Leaf morbidity statistics of *SlCSN5*-3 silenced tomatoes under gray mold disease stress for 120 h (inoculum: 4 × 10^6^/mL spore concentration). Error lines represent the standard error of the mean of the data from triplicate experiments. ** denotes *p* < 0.001 (*t*-test).

Control and *SlCSN5-3* silenced tomatoes were subjected to a gray mold disease stress treatment, and the phenotype and leaf morbidity of the tomatoes were observed at 120 h of the stress treatment. The results showed that *SlCSN5-3* silenced tomatoes were more severely ill compared with control tomatoes after 120 h of gray mold disease stress treatment (Figure 6A). Trypan blue results showed that the stained area of *SlCSN5-3* silenced tomato leaves was significantly larger than that of the control tomato leaves after 120 h of gray mold disease stress (Figure 6B). This suggests that more cells died in the diseased region of *SlCSN5-3* silenced tomato leaves. In addition, the statistical results of leaf morbidity at 120 h of gray mold disease stress treatment also showed that the leaf morbidity of *SlCSN5-3* silenced tomatoes was significantly higher than that of the control tomatoes (Figure 6C).

**Figure 7:**
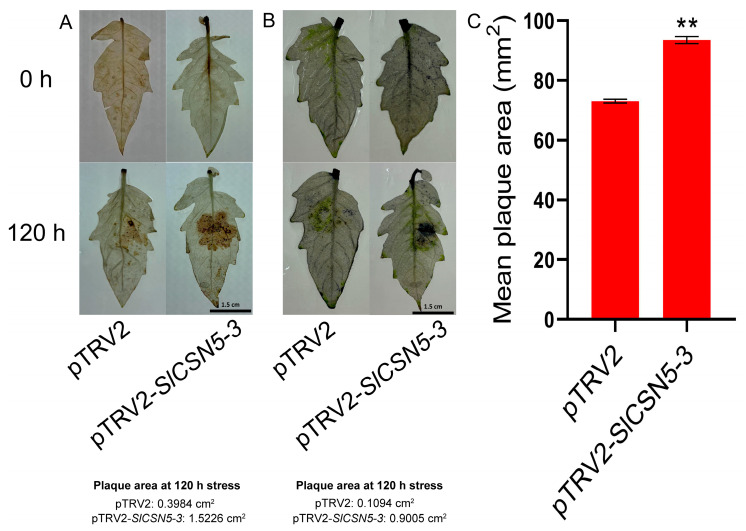
(**A**) DAB staining of *SlCSN5-3* silenced tomato leaves under gray mold disease stress (inoculum: 4 × 10^6^/mL spore concentration). Darker brown indicates more H_2_O_2_ accumulation. (**B**) NBT staining of *SlCSN5-3* silenced tomato leaves under gray mold disease stress (inoculum: 4 × 10^6^/mL spore concentration). Darker blue indicates more O_2_^−^ accumulation. (**C**) Mean diseased spot area of *SlCSN5-3* silenced tomato leaves under gray mold disease stress (inoculum: 4 × 10^6^/mL spore concentration). Error lines represent the standard error of the mean of the data from triplicate experiments. ** denotes *p* < 0.001 (*t*-test).

The DAB and NBT staining on the leaves of the control and *SlCSN5-3* silenced tomatoes after gray mold disease stress were performed to assess oxidative damage in the disease spot area. The results showed that the situ accumulation of H_2_O_2_ and O_2_^−^ in the diseased spot area of the *SlCSN5-3* silenced tomatoes was more than the control tomatoes (Figure 7A,B), which indicated that the oxidative damage of *SlCSN5-3* silenced tomato leaves was more serious. The average disease spot area of diseased leaves was also counted, which showed that the average disease spot area of diseased leaves in *SlCSN5-3* silenced tomatoes was significantly higher than that in control tomatoes (Figure 7C). These results indicate that silencing *SlCSN5-3* significantly reduces tomatoes’ resistance to gray mold disease.

### 3.10. SlCSN5-3 Positively Regulates Tomatoes’ Resistance to Gray Mold Disease by Affecting the JA Pathway

**Figure 8:**
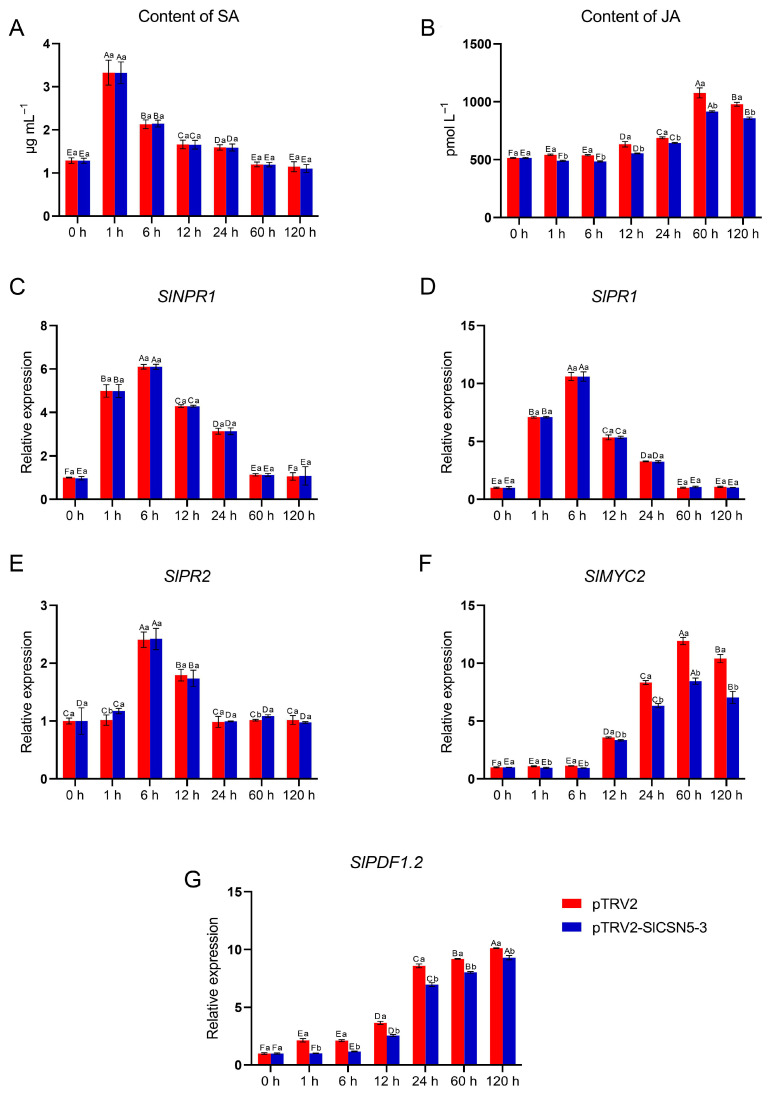
(**A**) The content of SA in the *SlCSN5-3* silenced tomatoes under gray mold disease stress. (**B**) The content of JA in the *SlCSN5-3* silenced tomatoes under gray mold disease stress. (**C**) The expression of SA pathway core transcription factor *SlNPR1* in the *SlCSN5-3* silenced tomatoes under gray mold disease stress. (**D**) The expression of SA pathway key disease-resistance gene *SlPR1* in the *SlCSN5-3* silenced tomatoes under gray mold disease stress. (**E**) The expression of SA pathway key disease-resistance gene *SlPR2* in the *SlCSN5-3* silenced tomatoes under gray mold disease stress. (**F**) The expression of JA pathway core transcription factor *SlMYC2* in the *SlCSN5-3* silenced tomatoes under gray mold disease stress. (**G**) The expression of JA pathway key disease-resistance gene *SlPDF1.2* in the *SlCSN5-3* silenced tomatoes under gray mold disease stress. Error lines represent the standard error of the mean of the data from triplicate experiments (*n* = 3). Capital letters represent the significance of the same plant at different times, according to Fisher’s LSD test at the 5% level of probability, and lowercase letters represent the significance of different plants at the same time, according to Fisher’s LSD test at the 5% level of probability.

The SA and JA content in control and *SlCSN5-3* silenced tomatoes under gray mold disease stress were examined, as well as the core transcription factors and core disease-resistance genes in the SA and JA pathways. The results showed that under gray mold disease stress, the SA content in the control tomatoes and *SlCSN5-3* silenced tomatoes increased significantly in the early stage of stress (0–1 h), dropped significantly after 6 h of stress, and basically dropped to the initial content after 60 h of stress (Figure 8A). The expression levels of *SlNPR1*, the core transcription factor of the SA pathway, and the key disease-resistance genes *SlPR1* and *SlPR2* were also increased in the early stage of disease and then decreased (Figure 8C–E). In addition, under gray mold disease stress, there was no significant difference in the SA content, expression of core transcription factors, and the key resistance genes of the SA pathway between the control tomatoes and the *SlCSN5-3* silenced tomatoes at the same time point, indicating that the SA pathway played a role in the resistance to gray mold disease in the early stage. However, this disease resistance regulation was not related to *SlCSN5-3*.

Under gray mold disease stress, the JA content in the control tomatoes and *SlCSN5-3* silenced tomatoes increased in the middle and late stages of disease (after 6 h), and the JA content in the *SlCSN5-3* silenced tomatoes was significantly lower than that in the control tomatoes at the same time points (Figure 8B). The core transcription factor *SlMYC2* and the key resistance gene *SlPDF1.2* of the JA pathway were also significantly increased in the middle and late stages of disease, and the expression levels of the above genes in the *SlCSN5-3* silenced tomatoes were lower than the control tomatoes at the same time point (Figure 8F,G), indicating that the JA-resistance pathway performed a function in the middle and late stages of gray mold disease and the *SlCSN5-3* expression was positively correlated with JA signaling.

**Figure 9:**
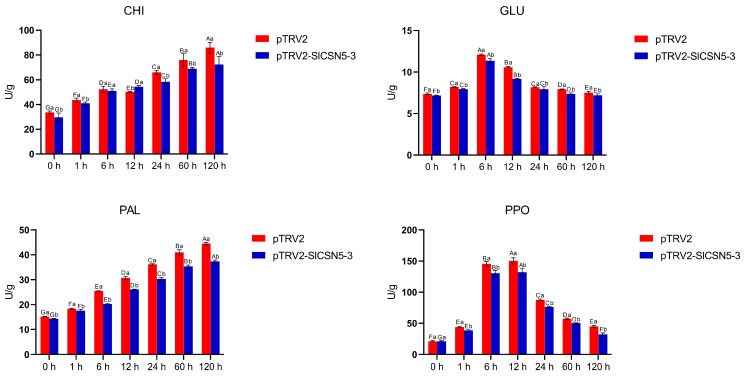
The activities of CHI, PAL, GLU, and PPO in the *SlCSN5-3* silenced tomatoes under *B. cinerea* stress. Error lines represent the standard error of the mean of the data from triplicate experiments (*n* = 3). Capital letters represent the significance of the same plant at different times, according to Fisher’s LSD test at the 5% level of probability, and lowercase letters represent the significance of different plants at the same time, according to Fisher’s LSD test at the 5% level of probability.

The activity of the disease resistance enzymes related to the JA pathway in the control and *SlCSN5-3* silenced tomatoes under gray mold disease stress was examined. The results showed that under gray mold disease stress, the enzyme activities of CHI and PAL increased significantly; GLU and PPO increased first and reached the peak at 6 h and 12 h after the stress, respectively, and then decreased. The activities of CHI, PAL, GLU, and PPO in the *SlCSN5-3* silenced tomatoes under gray mold disease stress were significantly lower than those in the control tomatoes (Figure 9). In conclusion, the expression of *SlCSN5-3* was positively correlated with the strength of the JA pathway and positively regulated the resistance of tomatoes to gray mold disease by affecting the expression of disease-resistance genes and enzymes through the JA pathway.

## 4. Discussion

Gray mold disease is a disease that often occurs in tomatoes and is caused by a fungus (*B. cinerea*) [21,22,23,24]. *B. cinerea* can infect tomatoes and other crops, causing losses to agricultural production [25,26,27]. In addition to regulating plant life activities by participating in the ubiquitination process, the COP9 signalosome subunit, CSN5, also regulates plant defense responses [28]. In this study, a total of three genes encoding the CSN5 protein in tomatoes were identified by bioinformatics methods (Table S2), and the chromosomal localization results showed that these three genes were distributed at both ends of the chromosome (Figure 1), indicating that the *CSN5* family genes may have high expression activity in tomato. The *CSN5* family genes are not uniformly distributed in chromosomes, which are present in most gene family studies [29,30,31,32,33]. The *CSN5* family genes identified in tomatoes all contain introns, and their encoded SlCSN5 proteins all contain only the conserved MPN_RPN11_CSN5 domain (Figure 2). The MPN domain is the core domain of the CSN5 protein [5], which indicates that our identification is accurate. Phylogenetic analysis showed that the CSN5 protein in tomatoes could be grouped into the same branch as the CSN5 protein in other plants, but the CSN5 protein in Arabidopsis was highly conserved. The collinearity analysis showed that the *SlCSN5* genes had no collinearity in tomato, and there was no synteny in the *CSN5* genes in tomatoes and Arabidopsis, but there was synteny in the *CSN5* genes in tomatoes and potatoes (Figure 3). It is possible that tomatoes and potatoes are closely related, and the CSN5 protein in Arabidopsis is highly conserved. The results of *cis*-acting elements showed that the promoter regions of the tomato *CSN5* family genes were rich in *cis-acting* elements related to stress response (Figure 4A). This suggests that there may be a correlation between the tomato *CSN5* family genes and stress.

When plants are exposed to external stress, the expression of their own genes changes to adapt to the stress [34,35,36]. By analyzing the transcriptome data of tomatoes under gray mold disease stress and qRT-PCR validation, the result showed that *SlCSN5-2* and *SlCSN5-3* were up-regulated under gray mold disease stress; this suggests that *SlCSN5-2* and *SlCSN5-3* may play a positive role in the tolerance process of tomatoes to gray mold disease. Although the expression levels of *SlCSN5-2* and *SlCSN5-3* eventually increased under gray mold disease stress, *SlCSN5-2* showed a transient down-regulation expression in the early stage of stress, while *SlCSN5-3* expression always increased, and the increase was significantly higher than that of *SlCSN5-2* (Figure 4B,C); therefore, *SlCSN5-3* was selected as a potential key gene for subsequent gene function studies. The *SlCSN5-3* was silenced in tomatoes to verify its function in tomatoes’ stress tolerance to gray mold disease. Under gray mold disease treatment, *SlCSN5-3* silenced tomatoes showed a larger diseased spot area and higher diseased leaf rate than the control tomatoes (Figure 6A,C and Figure 7C). In addition, other indicators attempted to assess the disease resistance of *SlCSN5-3* silenced tomatoes. Trypan blue cannot enter the living cells smoothly, due to the isolation of the cell membrane. When the isolation of the cell membrane is damaged after cell death, trypan blue can enter the cells and dye the cells blue [37,38,39]. When plants are stressed, they are locally accompanied by strong oxidative damage, which is reflected in the excessive accumulation of H_2_O_2_ and O_2_^−^ [40]. Therefore, trypan blue, DAB, and NBT staining were used to judge cell death and ROS accumulation in the leaves of *SlCSN5-3* silenced tomatoes under gray mold disease treatment to assess the disease resistance of *SlCSN5-3* silenced tomatoes. The results showed that *SlCSN5-3* silenced tomatoes had larger cell death areas and more in situ ROS accumulation in leaves than the control tomatoes (Figure 6B and Figure 7A,B), indicated that silencing *SlCSN5-3* reduced the resistance of tomatoes to gray mold disease.

The SA and JA signaling pathways are closely related to plant disease resistance [41,42,43]. To understand the interaction between *SlCSN5-3* and the SA and JA signaling in tomatoes, the SA and JA contents and the expression of the core genes of the SA and JA pathways in *SlCSN5-3* silenced tomatoes under gray mold disease treatment were examined. The results showed that under gray mold disease stress, the SA signal intensity increased in the early stage of stress, and the JA signal began to increase in the middle and late stages of stress, and there was antagonism between the SA and JA signals (Figure 8A,B), which was also found in previous research results; this antagonism implies that the SA pathway plays a role in disease resistance in the early stage of infection and is gradually replaced by the JA pathway [44,45]. In addition, JA signaling was significantly reduced in the *SlCSN5-3* silenced tomatoes compared to the control tomatoes at the same time points, suggesting that the *SlCSN5-3* is upstream of the JA signaling (Figure 8D,F,G). *B. cinerea* is a necrotrophic fungus that relies on dead host tissues for nutrition [46,47,48]. The MYC2 transcription factor is the core transcription factor in the JA pathway in plants [49]. When the JA signal is increased, MYC2 activates the expression of the disease resistance gene *PDF1.2* to fight necrotrophic fungus [50]. Therefore, it was hypothesized that *SlCSN5-3* may regulate tomatoes’ resistance to gray mold disease, depending on the JA pathway. In addition to the activation of disease-resistance genes, *SlCSN5-3*-dependent JA signaling also acts as a signaling molecule to mediate the enhancement of some disease-resistance enzyme activities (Figure 9). For example, CHI can be activated to accelerate the degradation of fungal cell walls after infection [51]. For PAL, which promotes lignin synthesis and thus improves cell wall strength, the enzyme activity was also up-regulated after infection to enhance the plant’s resistance to disease [52]. Under microbial stress, the up-regulated expression of GLU accelerates the decomposition of β-1, 3-glucanase in the fungal cell wall, and the up-regulated expression of PPO promotes the synthesis of quinones and enhances the broad-spectrum disease resistance of plants [53,54]. The increase in the activity of these resistance enzymes can significantly improve the disease resistance of plants. In conclusion, *SlCSN5-3* may positively regulate tomatoes’ resistance to gray mold disease by activating the expression of disease-resistance genes and enzymes that are dependent on the JA pathway (Graphical Abstract). This study deepened the theory of plants’ disease-resistance regulation, and future work will further explore whether SlCSN5-3 is involved in the regulation of ubiquitination.

## 5. Conclusions

A total of three *CSN5* family genes were identified in tomatoes’ SL4.0 genome and systematic bioinformatics analysis was performed. The expression of *SlCSN5-2* and *SlCSN5-3* increased after gray mold disease treatment. *SlCSN5-3* positively regulates tomatoes’ resistance to gray mold disease through the JA pathway. This study provides a new idea for tomato disease resistance breeding in the future.

## Data Availability

If required, please contact the corresponding author and data will be provided.

## Supplementary Materials

The following supporting information can be downloaded at: https://www.mdpi.com/article/10.3390/biology14121635/s1. Table S1: The primers used in this study; Table S2: Members of the tomato *CSN5* gene family and physicochemical properties of the encoded proteins based on SL4.0 annotation.

## Abbreviations

The following abbreviations are used in this manuscript:

CSN	Constitutive photomorphogenesis 9
JA	Jasmonic acid
MPN	Mprl-Padl-N-teminal
eIF3	Eukaryotic initiation factor 3
UPS	Ubiquitin/26S protease system
CDS	Coding sequence
HMM	Hidden Markov model
VIGS	Virus-induced gene silencing
DAB	Diaminobenzidine
NBT	Nitrotetrazolium blue
CHI	Chitinase
PAL	Phenylalanine ammonia lyase
GLU	β-1,3-glucanase
PPO	Polyphenol oxidase
SA	Salicylic acid
UTR	Untranslated region
