# Supplementary material for: COP9 Signalosome Subunit SlCSN5-3 Positively Regulates Resistance to Gray Mold Disease in Tomato (Solanum lycopersicum) Through Jasmonic Acid Pathway
Source: Biology (Basel). 2025 Nov 21;14(12):1635. doi: 10.3390/biology14121635 (PMC12729962; doi:10.3390/biology14121635)
Supplement: Supplementary file 1 [file biology-14-01635-s001.zip › biology-3985164-supplementary.pdf]

Table S1 The primers used in this study

Primer Name	Sequence
qPCR-SlCSN5-1-F	TGCCTCAGAGTGGTACTGGT
qPCR-SlCSN5-1-R	GAAAGCCAGCAGCCAAATCC
qPCR-SlCSN5-2-F	AGACGAAGTTTCAGCGGGAG
qPCR-SlCSN5-2-R	CCTGCATGAGTCCCATGACC
qPCR-SlCSN5-3-F	CCATTCACGTTTTGGGCACT
qPCR-SlCSN5-3-R	AACCTGCGACATTAAGCCAT
qPCR-β-actin-F	ACAACTTTCCAACAAGGGAAGAT
qPCR-β-actin-R	TGTATGTTGCTATTCAGGCTGTG
VIGS-CSN5-3-F	agtggtctctgtccagtcctATGGACGCTCTGAATTCTTACG
VIGS-CSN5-3-R	ggtctcagcagaccacaagtCGTCTTACCTTGCATTAGTCCCA
qPCR-SlMYC2-F	CTTCGGGCATGAAGTCAGG
qPCR-SlMYC2-R	TTCGCTGGCTTTCTACCTCG
qPCR-SlPDF1.2-F	CACTTCACAAATGTCGATCCG
qPCR-SlPDF1.2-R	AGCCAAATCCAATGCAGTCTC
qPCR-SlNPR1-F	GGGAAAGATAGCAGCACG
qPCR-SlNPR1-R	GTCCACACAAACACACACATC
qPCR-SlPR1-F	GATGTGGGACGATGAGAAGCAATG
qPCR-SlPR1-R	GTTGCATCGAACCCTAGCACAACCT
qPCR-SlPR2-F	CAGATTTCACTTCCGTATGCTCTT
qPCR-SlPR2-R	CCATCCACTCTCTGACACAACTAT

Table S2 Members of the tomato *CSN5* gene family and physicochemical properties of the encoded proteins based on SL4.0 annotation

Number	Gene Name	Gene ID	Chr	Start	End	Number of amino acids	pI	Molecular weight
1	*SlCSN5-1*	*Solyc04g079200.3.1*	04g	61724496	61729729	312	6.17	34708.85
2	*SlCSN5-2*	*Solyc06g073150.3.1*	06g	42729710	42734560	367	5.06	40835.84
3	*SlCSN5-3*	*Solyc11g017300.2.1*	11g	8261237	8266484	367	4.99	40835.8
